# The first occurrence of a CTX-M ESBL-producing *Escherichia coli* outbreak mediated by mother to neonate transmission in an Irish neonatal intensive care unit

**DOI:** 10.1186/s12879-016-2142-6

**Published:** 2017-01-05

**Authors:** Ciara O’Connor, Roy K. Philip, John Kelleher, James Powell, Alan O’Gorman, Barbara Slevin, Neil Woodford, Jane F. Turton, Elaine McGrath, Cathriona Finnegan, Lorraine Power, Nuala H. O’Connell, Colum P. Dunne

**Affiliations:** 1 Department of Clinical Microbiology, University Hospital Limerick, Limerick, Ireland; 2 Centre for Interventions in Infection, Inflammation and Immunity (4i), Graduate Entry Medical School, University of Limerick, Limerick, Ireland; 3 Department of Paediatrics, University Maternity Hospital Limerick, Limerick, Ireland; 4 Infection Prevention and Control Team, University Hospital Limerick, Limerick, Ireland; 5 Antimicrobial Resistance and Healthcare Associated Infections (AMRHAI) Reference Unit, Public Health England, London, UK; 6 Antimicrobial Resistance and Microbial Ecology Group, School of Medicine, National University of Ireland, Galway, Ireland

**Keywords:** ESBL, Outbreak, *Escherichia coli*, Ireland, CTX-M, NICU

## Abstract

**Background:**

*Escherichia coli (E. coli)* comprise part of the normal vaginal microflora. Transfer from mother to neonate can occur during delivery resulting, sometimes, in neonatal bacterial disease. Here, we aim to report the first outbreak of CTX-M ESBL-producing E. coli with evidence of mother-to-neonate transmission in an Irish neonatal intensive care unit (NICU) followed by patient-to-patient transmission.

**Methods:**

Investigation including molecular typing was conducted. Infection was defined by clinical and laboratory criteria and requirement for antimicrobial therapy with or without positive blood cultures. Colonisation was determined by isolation without relevant symptoms or indicators of infection.

**Results:**

Index case was an 8-day-old baby born at 34 weeks gestation who developed ESBL-producing *E. coli* infections at multiple body sites. Screening confirmed their mother as colonised with ESBL-producing *E. coli.* Five other neonates, in the NICU simultaneously with the index case, also tested positive. Of these, four were colonised while one neonate developed sepsis, requiring antimicrobial therapy. The second infected neonate’s mother was also colonised by ESBL-producing *E. coli.* Isolates from all eight positive patients (6 neonates, 2 mothers) were compared using pulsed-field gel electrophoresis (PFGE). Two distinct ESBL-producing strains were implicated, with evidence of transmission between mothers and neonates for both strains. All isolates were confirmed as CTX-M ESBL-producers. There were no deaths associated with the outbreak.

**Conclusions:**

Resources were directed towards control interventions focused on hand hygiene and antimicrobial stewardship, which ultimately proved successful. Since this incident, all neonates admitted to the NICU have been screened for ESBL-producers and expectant mothers are screened at their first antenatal appointment. To date, there have been no further outbreaks.

## Background

*Escherichia coli (E. coli)* comprise part of the normal vaginal microflora. Vertical transfer from mother-to-neonate can occur during delivery [1, 2] resulting, sometimes, in severe neonatal bacterial disease [3]. Extended-spectrum beta-lactamases (ESBLs) are plasmid-borne beta-lactamases (such as TEM-, SHV-, OXA- and CTX-M types) capable of hydrolysing and inactivating beta-lactam antimicrobials with an oxyimino side chain, e.g., cephalosporins (cefotaxime, ceftriaxone, ceftazidime) and the oxyimino-monobactam (aztreonam) [4]. First reported in 1989, CTX-M enzymes have represented the most prevalent ESBL-type worldwide since 2000 [5, 6]. In 2013, it was reported that incidence of community-associated infections caused by CTX-M ESBL-producing bacteria, particularly urinary tract infections in women of child-bearing age, is increasing [7]. ESBL-producing *E. coli* have been reported as a cause of neonatal sepsis and meningitis [8] and mother-to-neonate transmission of ESBL producers has been previously described [9]. The gastrointestinal tract of infected or colonised patients, of all age groups, is the most frequently-reported reservoir of ESBL-producing organisms, and studies have shown that transient carriage of ESBL-producing organisms on the hands of healthcare workers [10] or on artificial nails [11] may also facilitate transmission.

Neonatal intensive care units (NICUs) have been described as an interface between the hospital and the community due to the possibility of parents, while providing daily care for their infants, introducing community-associated multi-drug resistant organisms (MDROs) including ESBL-producers [12, 13]. NICU stays have become prolonged due to advances in modern medicine, with duration of hospitalisation inversely related to gestational age and with increased risk of hospital-acquired infection [14]. Specifically, risk factors associated with colonisation or infection by ESBL-producers in NICUs include low gestational age, an immature immune system, low birth weight, care in incubators, exposure to third-generation cephalosporins [15, 16] and contaminated breastmilk [17]. Septicaemia due to ESBL-producing organisms has been associated with a significantly increased mortality rate compared to non-ESBL-producing isolates [18]. In general, chemotherapeutic options for dealing with ESBL-related infections are limited, and that challenge is compounded by restrictive prescribing for neonates due to the potential for adverse side-effects. Therefore, when they occur, NICU-associated nosocomial infections increase hospital costs substantially, with the potential to prolong hospitalisation considerably, and are responsible for 50% of deaths that occur beyond two weeks of age [19]. In addition, there may be disruption of healthcare services due to stringent infection control measures such as restriction of admissions or ward closures.

In this report, we describe the occurrence and outcomes of the first ESBL-producing *E. coli* outbreak in an Irish NICU. In particular, we detail the infection prevention and control interventions that successfully brought the outbreak to an end.

## Methods

### Setting

The University Maternity Hospital Limerick in Ireland (UMHL) is a tertiary referral centre and includes a NICU with a total of 19 cots. The catchment population of UMHL is approximately 400,000. In the twelve months prior to the outbreak, there were 4905 live births and 909 NICU admissions (of all gestational ages). At the time of this outbreak, the NICU had one intensive care ward consisting of four neonatal intensive care cots, five high dependency cots and two isolation rooms. Two intermediate care rooms, separated from the intensive care ward, contained a further 10 cots. With respect to prevention of nosocomial infection, the NICU intensive care ward provided four washing stations, alcohol hand gels at each bedspace, and a nurse to patient ratio of 1:1 for ICU category cots and 1:2 or 1:3 (depending on staffing levels) for the remainder. A weekly multi-disciplinary NICU ward-round was performed.

### Index case identification

The index case for this outbreak was an infant born (at 34 weeks gestation) in week 11 2013 via spontaneous vaginal delivery. Whilst in the NICU swab cultures from separate areas on the body were positive for ESBL-producing *E. coli* resistant to co-amoxiclav, ceftriaxone, aztreonam, ciprofloxacin, gentamicin and piperacillin/tazobactam; sensitive to chloramphenicol, amikacin and meropenem. The infant was placed in an isolation room with contact precautions, chloramphenicol eye drops and IV meropenem (dosed as per weight) administered for seven days. They was discharged home on day 17 after birth without need for further antimicrobials.

### Infection control interventions

Contact tracing of all inpatients who may have been in contact with the index case while in NICU was conducted using urine samples and/or rectal swabs, one sample for each neonate or mother was analysed depending on what was most easily obtained. With consent, the mother of the index case and mothers of subsequently positive neonates were screened for the presence of ESBLs using rectal swabs, high vaginal swabs and mid-stream urine samples. Following a review of related literature, and to be prudent, a decision was made to close the NICU to new admissions from week 12 2013, with exception of emergencies, and visiting was restricted to parents of inpatients only awaiting the results of contact tracing.

Following confirmatory cultures results, all infected or colonised neonates were barrier nursed by personnel wearing disposable gowns and gloves. In addition, a restriction was placed on the prescription of third generation cephalosporins. Due to the availability of only two isolation rooms in the NICU, the index case and one other neonate infected by ESBL-producers were isolated. The remaining colonised neonates were cohorted in incubators in the main NICU ward, with dedicated single-patient equipment. Given physical environmental constraints, it was not possible to increase the space between cots. Neonates who were fit for discharge were cohorted to a single post-natal maternity ward to minimise cross-transmission.

The isolation of an ESBL-producing *E. coli* from a NICU inpatient triggered initiation of the hospital’s outbreak management protocol, which involved meeting with all key stakeholders, including executive management, nursing administration, infection prevention and control, consultant microbiologists, laboratory managers, bed management, hygiene services, communications team and NICU clinical director. Following a review of related literature, and to be prudent, a decision was made to close the NICU to new admissions, with exception of emergencies, and visiting was restricted to parents of inpatients only. Information leaflets regarding outbreak risks and management were distributed to visiting parents. Appropriate public communication and a press statement were issued by the Clinical Director. Arrangements were made for antenatal inpatients whose neonates might require NICU admission to be referred to other maternity hospitals in Ireland. Empiric IV meropenem (dosed as per weight) was administered for any infant demonstrating signs of sepsis, pending microbiology analysis of urine or rectal swabs. All infected or colonised neonates were barrier nursed by personnel wearing disposable gowns and gloves. In addition, a restriction was placed on the prescription of third generation cephalosporins

With respect to hygiene, enhanced cleaning of the NICU was instigated in parallel with increased auditing. This involved twice-daily cleaning of affected areas and incubators with detergent. Air sampling and environmental sampling were not performed. An intensive targeted educational programme focussed on standard precautions, particularly hand hygiene compliance and on modes of transmission of ESBL-producing *E. coli* transmission was provided to all clinical and administrative staff. Screening of staff for carriage of ESBL-producing *E. coli* was not conducted but instead it was considered of important to put emphasis on zero tolerance to poor compliance with the World Health Organisation’s “5 moments for hand hygiene”. Hand hygiene audits were performed with greater frequency in affected areas, which involved twice weekly observational audits at ward level. The last positive isolate was identified in week 13 2013. The NICU re-opened later in week 13 2013 and weekly multi-disciplinary meetings were held until week 18 2013 to discuss and implement hygiene recommendations, at which point the outbreak was declared over. The last known neonate involved in the outbreak was discharged in week 16 2013.

### Microbiological and molecular detection of ESBL-producing *E. coli*

At the time of the outbreak, the routine ESBL screening policy targeted weekly screening of high-risk neonates identified as such by their managing Consultant Neonatologists (but broadly categorised as premature or in NICU for other than short term stay) and their mothers.

Screening specimens were cultured using ChromID™ ESBL agar (bioMérieux, Marcy l’Ȇtoile, France) and incubated at 37 °C aerobically for 18–24 h. The colour code guide provided by the manufacturer was followed for review of any colonies identified; pink/brown = presumptive *E. coli*, green/blue = presumptive *Klebsiella* species, white = other Enterobacteriaceae. All colonies were identified using MALDI-ToF MS (Bruker Daltonics, Bremen, Germany) as described previously [20]. Confirmatory testing was performed by disk diffusion on all organisms that warranted further investigation (Thermo Scientific™ Oxoid™ Disks) using a disc dispenser; cefoxitin 30 μg (FOX), cefapime 30 μg (FED), ceftazidime 30 μg (CAZ) & ceftazidime-clavulanic acid 30/10 μg (CAZCV), cefotaxime 30 μg (CTX) & cefotaxime-clavulanic acid 30/10 μg (CTXCV), Muller Hinton agar, 0.5 McFarland inoculum; 35 +/−2 °C, ambient air, 16–28 h. Following incubation, the zone sizes of the cephalosporin disc to that of a cephalosporin plus clavulanic acid combination disc were compared to determine ESBL status. Criteria for positive ESBL disc confirmatory testing on Enterobacteriaceae: a ≥5 mm increase in zone diameter for either antimicrobial agent tested in combination with clavulanic acid versus its zone when tested alone. Criteria for negative ESBL disc confirmatory testing on Enterobacteriaceae: zone sizes for both cephalosporin and cephalosporin in combination with clavulanic acid that is equal or show no greater difference in diameter than +/− 2 mm. Criterion for inconclusive ESBL disc confirmatory testing: difference between matched discs was >2 but <5 mm. In 2013, our protocols dictated that any inconclusive disk diffusion result, would warrant further confirmatory testing via Etest (bioMérieux, Marcy l’Ȇtoile, France). No isolate from this outbreak required further testing via Etest.

The genetic relationships between the ESBL-producing *E. coli* isolates were determined by pulsed-field gel electrophoresis (PFGE) of *Xba*I-digested genomic DNA at the Antimicrobial Resistance and Healthcare Associated Infections (AMRHAI) Reference Unit, Public Health England, London, UK. Electrophoresis was performed on a Bio-Rad CHEF DRII apparatus at 6 V cm^−1^ for 30 h at 12 °C with ramping times of 5 s to 35 s. CTX-M subtyping was possible on all but one of the isolates subsequent to the outbreak.

### Processing of breast milk specimens

Routine breast milk testing involved incubating undiluted samples spread on blood agar (5-10% CO_2_) for up to 48 h, blood agar with metronidazole for up to 48 h anaerobically, and MacConkey Agar for up to 48 h aerobically.

## Results

### Epidemiological features of the outbreak

Following detection of the index case (neonate two, Table 1), between weeks 12 and 13 2013, following a comprehensive screening seven additional screens of two NICU mothers and five neonates proved positive (Table 1). During this outbreak, infection was defined by clinical and laboratory criteria and requirement for antimicrobial therapy, while colonisation was defined by the absence of relevant symptoms. In total, during the outbreak, 86 ESBL screens from 42 individuals were performed. Of these, specimens from six neonates were positive for ESBL-producing *E. coli*: two represented infection and four represented colonisation. The mean gestational age was 33 weeks (range 28 to 36 weeks). There were no bacteraemias due to ESBL-producers.

**Table 1:** Clinical characteristics of the neonates affected

Neonate	Gestational age at birth (weeks)	Delivery^a^	ESBL Source	ESBL resistance mechanism	Days in NICU prior to positive result	Infected/colonised	Feeding^b^	Status at time of discharge from NICU	Repeat ESBL testing post outbreak
1	28 + 2	CS	Rectal	Unknown	46	Colonised	EBM	Alive	Not performed
2 (index case)	34 + 5	VD	Right buttock, right eye, urine, rectal	CTX-M	8	Infected^c^	EBM	Alive	Not performed
3	36 + 3	CS	Rectal	CTX-M	8	Colonised	EBM	Alive	Not performed
4	36 + 3	CS	Rectal	CTX-M	8	Colonised	EBM	Alive	Not performed
5	35 + 4	CS	Rectal	CTX-M	7	Colonised	Formula	Alive	Not performed
6	30 + 6	VD	Rectal, urine	CTX-M	6	Infected^d^	EBM	Alive	ESBL positive rectal swab September 2013

^a^CS: caesarean section, VD: vaginal delivery

^b^EBM: expressed breast milk

^c^Ophthalmic, skin/soft tissue infection

^d^Urosepsis

The index case neonate’s mother was informed and agreed to participate in screening. A rectal swab, a mid-stream urine and a sample of expressed breast milk all tested positive for ESBL-producing *E. coli,* as shown in Table 2, with the same antibiogram as the isolate from her neonate (detailed earlier). A high vaginal swab was negative for the bacterium. This isolation of an ESBL-producing *E. coli* was the first such result for the patient who had never before had a culture-positive urine test.

**Table 2:** Microbiology culture results of mothers involved

	High vaginal swab	Rectal swab	Mid-stream urine	Expressed breast milk	ESBL resistance mechanism
Mother of neonate 1	negative	negative	negative	negative	not tested
Mother of neonate 2 (index case)	negative	ESBL positive	ESBL positive	ESBL positive	CTX-M
Mother of neonates 3&4	negative	not tested	not tested	not tested	not tested
Mother of neonate 5	negative	negative	negative	not tested	not tested
Mother of neonate 6	negative	ESBL positive	ESBL positive	negative	CTX-M

**Fig. 1:**
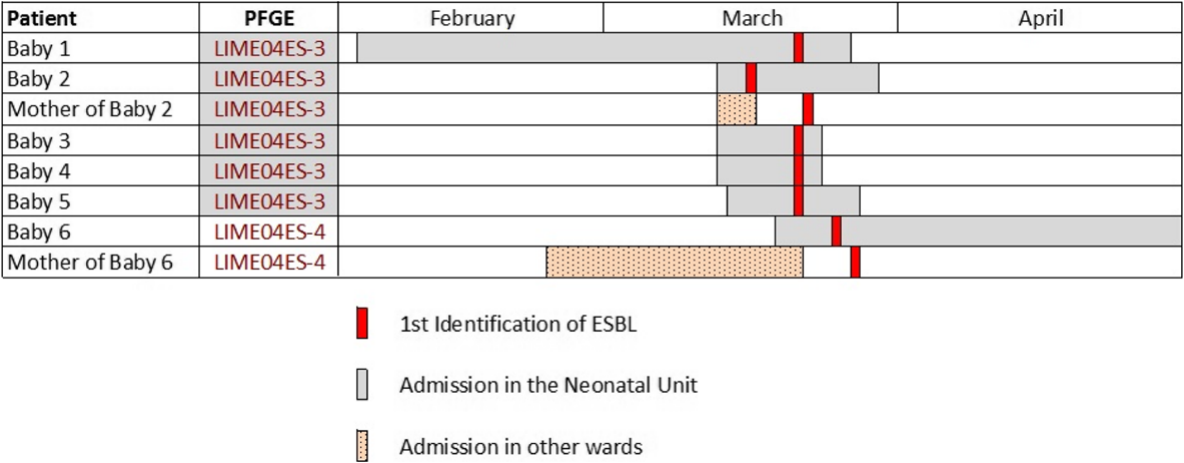
Visual depiction of the timelines associated with this occurrence

One further neonate (neonate six, Table 1) who was in the NICU at the time of the outbreak (Fig. 1) developed urosepsis, eight days after the first detection of an ESBL-producer in the NICU. While two blood specimens proved negative, ESBL-producing *E. coli* were confirmed from a urine sample and from a rectal swab. An identical antibiogram to that of the index case neonate was noted. The baby was treated with IV meropenem (dosed as per weight). This neonate’s mother was screened whereupon a high vaginal swab and a sample of expressed breast milk cultured negative, but a rectal swab and a mid-stream urine sample were positive for ESBL-producing *E. coli* demonstrating the same antibiogram as previously found. Again, this second mother had no previous documented urinary tract infections and had never before had a culture-positive urine test.

Of the remaining four neonates, all were clinically stable, underwent weekly surveillance rectal cultures until discharged from the NICU, and did not require treatment with antimicrobials. All mothers of colonised infants underwent screening to determine colonisation or infection and were found to be negative for ESBL-producers.

### Molecular characteristics and antibiogram of the outbreak

All ESBL isolates identified during this outbreak had an identical antibiogram regardless of culture source: resistant to ampicillin/amoxicillin, co-amoxiclav, ceftriazone, aztreonam, ciprofloxacin, gentamicin, cefuroxime, piperacillin/tazobactam, tri/sulfamethoxazole, while susceptible to amikacin, chloramphenicol, ertapenem, and meropenem. Following sequencing, all were found to bear CTX-M-15 and OXA-1.

**Fig. 2:**
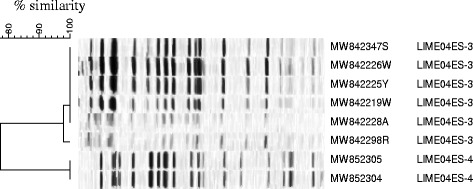
PFGE dendrogram illustrating likely cross-transmission, by strain LIME04ES-3, between the index case (MW8423247S) and his mother (MW842298R) and four other neonates. The second strain (LIME04ES-4) was isolated from another neonate (MW852305), who developed an ESBL-related infection requiring treatment, and his mother (MW852304)

Following PFGE analysis (see Fig. 2), strain designated LIME04ES-3 was found to be shared between the index case, their mother and four other neonates, indicating cross-transmission and was determined to be ST131. A second strain (LIME04ES-4), determined to be ST1284, was found in both the second infected neonate and their mother.

## Discussion

Mother-to-neonate transmission of ESBL-producing *E. coli* in a European NICU has been described previously, with a 2010 report from Switzerland of an outbreak that began with transmission from a mother to her newborn twins during vaginal delivery with subsequent spread facilitated by health care workers [21]. It is noteworthy that a 2013 report traced another NICU outbreak to ESBL-producers originating with a neonate born via caesarean section and exclusively formula-fed [22]. Taken together, these reports involving both delivery methods exemplify the potential risk of infection and/or spread regardless of mode of delivery [23].

The is first occurrence of an ESBL-producing *E. coli* outbreak in a neonatal intensive care unit in Ireland mediated by mother-to-neonate transmission that has been confirmed by molecular analysis. The index case’s mother had a rectal swab and a mid-stream urine sample that cultured positive for ESBL-producing *E. coli* although the organism was not detected from the high vaginal swab. Consensus was that the likely route of transmission was vertical from the mother’s colonised perianal area during delivery. The second ESBL strain (LIM04ES-4), from a separate neonate (neonate six) and their mother, was again thought to have been transmitted in the same manner from mother to child, who had positive results from a rectal swab and mid-stream urine.

Neither staff nor environmental screening were performed at the time of the outbreak. The focus was instead directed towards coordinating and managing the NICU closure, staff education regarding hand hygiene & transmissibility of ESBL-producers, antimicrobial stewardship for NICU prescribers, and audit of hand & environmental hygiene. In contrast, a Greek outbreak of SHV-5-producing *Klebsiella pneumoniae* in 2012, involving 13 infected and three colonised neonates [24], employed staff and environmental screening whereby both staff and environmental sites were found to be negative with no specific case identified as being the origin of the outbreak. They further implemented the same outbreak management steps as were followed in our outbreak, with the exception of NICU closure. Without data regarding environmental or staff screening, we believed that the unit closure was key to reducing the duration of the outbreak in allowing deep cleaning to occur, reducing incubator/cot occupancy, lowering the nurse:infant ratio, reducing the throughput of clinical staff into the NICU and curtailing the number of antimicrobials in use. Additional sequential control measures added value to our outbreak control including cohort nursing and a ‘look back’ to identify the outbreak boundaries, as well as determining recommendations for adherence regarding: shared use of communal breast pumps; separation of clean and used equipment; physical environment cleaning; sufficient staffing with hygiene service and cleaning personnel; and ensuring of sufficient supply of sanitiser-filled alcohol hand dispensers.

In the USA, active surveillance strategies have been adopted by many NICUs to detect infants colonized with antibiotic-resistant organisms albeit that the yield, risks, benefits and costs of different strategies have not been fully evaluated [25]. It is estimated that 21% of UK NICUs undertake routine faecal/rectal swabbing for ESBL-producing Enterobacteriaceae [26], but there is currently no consensus in Europe with regard to screening in NICUs [27]. Irish data regarding screening remain unknown and a study that reviewed the practices of NICUs from both the UK and Ireland concluded that NICUs *“currently lack systematic neonatal infection surveillance”* [28, 29]*.* A 2014 Swedish study reported that adopting once-a-week screening of all neonates can reduce time from admission to detection by eight days and lead to a substantial reduction in secondary cases and clinical infections [30]. Researchers in the USA determined that the rate of colonization by antibiotic-resistant bacteria was low, particularly in neonates <7 days old and have recommended that future studies should examine the safety of targeted surveillance strategies focused on older infants [25]. As a result of the outbreak reported here, and as part of our active surveillance programme, all neonates admitted currently to our NICU are screened for ESBL-producers via culture of a urine sample or stool sample or rectal swab on arrival. The screening culture protocol is described in the methods section. Thereafter, diagnostic microbiology analysis is performed if deemed clinically necessary.

Similarly, there is also no consensus with regard to ESBL screening of expectant mothers and, given the mother-to-neonate transmission identified in this Irish outbreak, this may be an area for enhanced surveillance with a view to improvement in reduction of neonatal risk. As a consequence of this outbreak, our current practice (similar to an approach being adopted as prudent in Norway [31]) is that all pregnant women presenting at their first antenatal appointment have ESBL screening performed via a urine sample, albeit resulting perhaps in the screening of inappropriately large numbers of healthy young women with a recognised low positive predictive value.

## Conclusions

In our setting, which may be comparable to many others, once an outbreak was declared, containment and control were achieved via timely closure of the unit to new admissions, staff education, strict adherence to hand hygiene measures with frequent auditing of staff compliance, cohorting infected neonates, screening of all inpatients, enhanced deep cleaning of all equipment within the NICU and utilising of an outbreak management team. We benefited from modification of the antimicrobial policy to move from broad-spectrum antibiotics to those that the problematic strain was susceptible to. A multidisciplinary approach was employed incorporating frequent communication with parents while visitor restrictions were enforced. With a deficit in national or international screening guidelines, in the context of a rising national ESBL-producer prevalence in Ireland, our screening practices as described here continue. To date, we have not had further ESBL outbreaks on any of our antenatal or postnatal wards due to appropriate early infection control management of newly identified ESBL patients. We hope that others may learn from our experience of successfully managing a neonatal ESBL outbreak.
